# Current Scenario and Future Prospects of Endophytic Microbes: Promising Candidates for Abiotic and Biotic Stress Management for Agricultural and Environmental Sustainability

**DOI:** 10.1007/s00248-023-02190-1

**Published:** 2023-03-14

**Authors:** Uttpal Anand, Tarun Pal, Niraj Yadav, Vipin Kumar Singh, Vijay Tripathi, Krishna Kumar Choudhary, Awadhesh Kumar Shukla, Kumari Sunita, Ajay Kumar, Elza Bontempi, Ying Ma, Max Kolton, Amit Kishore Singh

**Affiliations:** 1 Zuckerberg Institute for Water Research, The Jacob Blaustein Institutes for Desert Research, Ben-Gurion University of the Negev, Sede Boqer Campus, 8499000 Midreshet Ben-Gurion, Israel; 2 French Associates Institute for Agriculture and Biotechnology of Drylands, The Jacob Blaustein Institutes for Desert Research, Ben-Gurion University of the Negev, Sde Boker Campus, 8499000 Midreshet Ben-Gurion, Israel; 3 Department of Botany, K.S. Saket P.G. College, Ayodhya affiliated to Dr. Rammanohar Lohia Avadh University, Ayodhya, 224123 Uttar Pradesh India; 4 Department of Molecular and Cellular Engineering, Jacob Institute of Biotechnology and Bioengineering, Sam Higginbottom University of Agriculture, Technology and Sciences, Prayagraj, 211007 Uttar Pradesh India; 5 Department of Botany, Mahila Mahavidyalaya, Banaras Hindu University, Varanasi, 221005 Uttar Pradesh India; 6 Department of Botany, Deen Dayal Upadhyay Gorakhpur University, Gorakhpur, Uttar Pradesh 273009 India; 7 Department of Postharvest Science, Agricultural Research Organization, The Volcani Center, P.O. Box 15159, 7505101 Rishon, Lezion Israel; 8 INSTM and Chemistry for Technologies Laboratory, University of Brescia, Via Branze 38, 25123 Brescia, Italy; 9 Centre for Functional Ecology, Department of Life Sciences, University of Coimbra, Calçada Martim de Freitas, 3000-456 Coimbra, Portugal; 10 Department of Botany, Bhagalpur National College (A constituent unit of Tilka Manjhi Bhagalpur University), Bhagalpur, 812007 Bihar India

**Keywords:** Endophytes, Bioactive secondary metabolites, Biotic and abiotic stress, Biocontrol, Phytoremediation, Bioaccumulation

## Abstract

Globally, substantial research into endophytic microbes is being conducted to increase agricultural and environmental sustainability. Endophytic microbes such as bacteria, actinomycetes, and fungi inhabit ubiquitously within the tissues of all plant species without causing any harm or disease. Endophytes form symbiotic relationships with diverse plant species and can regulate numerous host functions, including resistance to abiotic and biotic stresses, growth and development, and stimulating immune systems. Moreover, plant endophytes play a dominant role in nutrient cycling, biodegradation, and bioremediation, and are widely used in many industries. Endophytes have a stronger predisposition for enhancing mineral and metal solubility by cells through the secretion of organic acids with low molecular weight and metal-specific ligands (such as siderophores) that alter soil pH and boost binding activity. Finally, endophytes synthesize various bioactive compounds with high competence that are promising candidates for new drugs, antibiotics, and medicines. Bioprospecting of endophytic novel secondary metabolites has given momentum to sustainable agriculture for combating environmental stresses. Biotechnological interventions with the aid of endophytes played a pivotal role in crop improvement to mitigate biotic and abiotic stress conditions like drought, salinity, xenobiotic compounds, and heavy metals. Identification of putative genes from endophytes conferring resistance and tolerance to crop diseases, apart from those involved in the accumulation and degradation of contaminants, could open new avenues in agricultural research and development. Furthermore, a detailed molecular and biochemical understanding of endophyte entry and colonization strategy in the host would better help in manipulating crop productivity under changing climatic conditions. Therefore, the present review highlights current research trends based on the SCOPUS database, potential biotechnological interventions of endophytic microorganisms in combating environmental stresses influencing crop productivity, future opportunities of endophytes in improving plant stress tolerance, and their contribution to sustainable remediation of hazardous environmental contaminants.

**Graphical Abstract:**

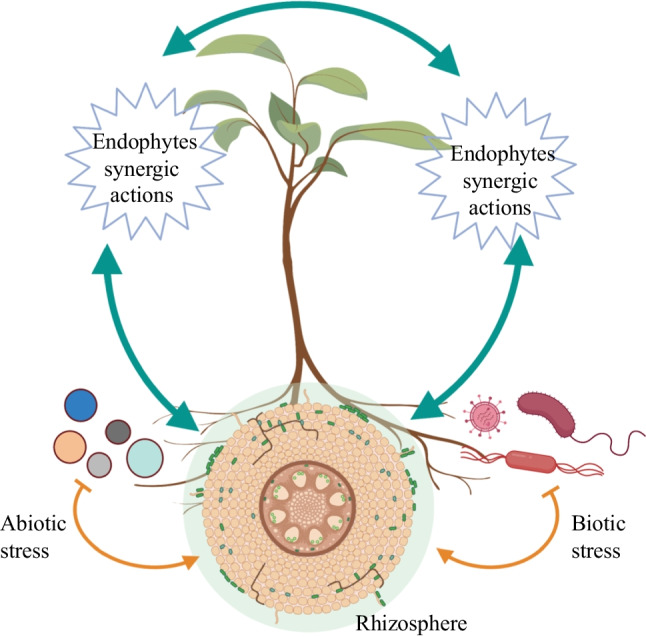

## Introduction

**Fig. 1:**
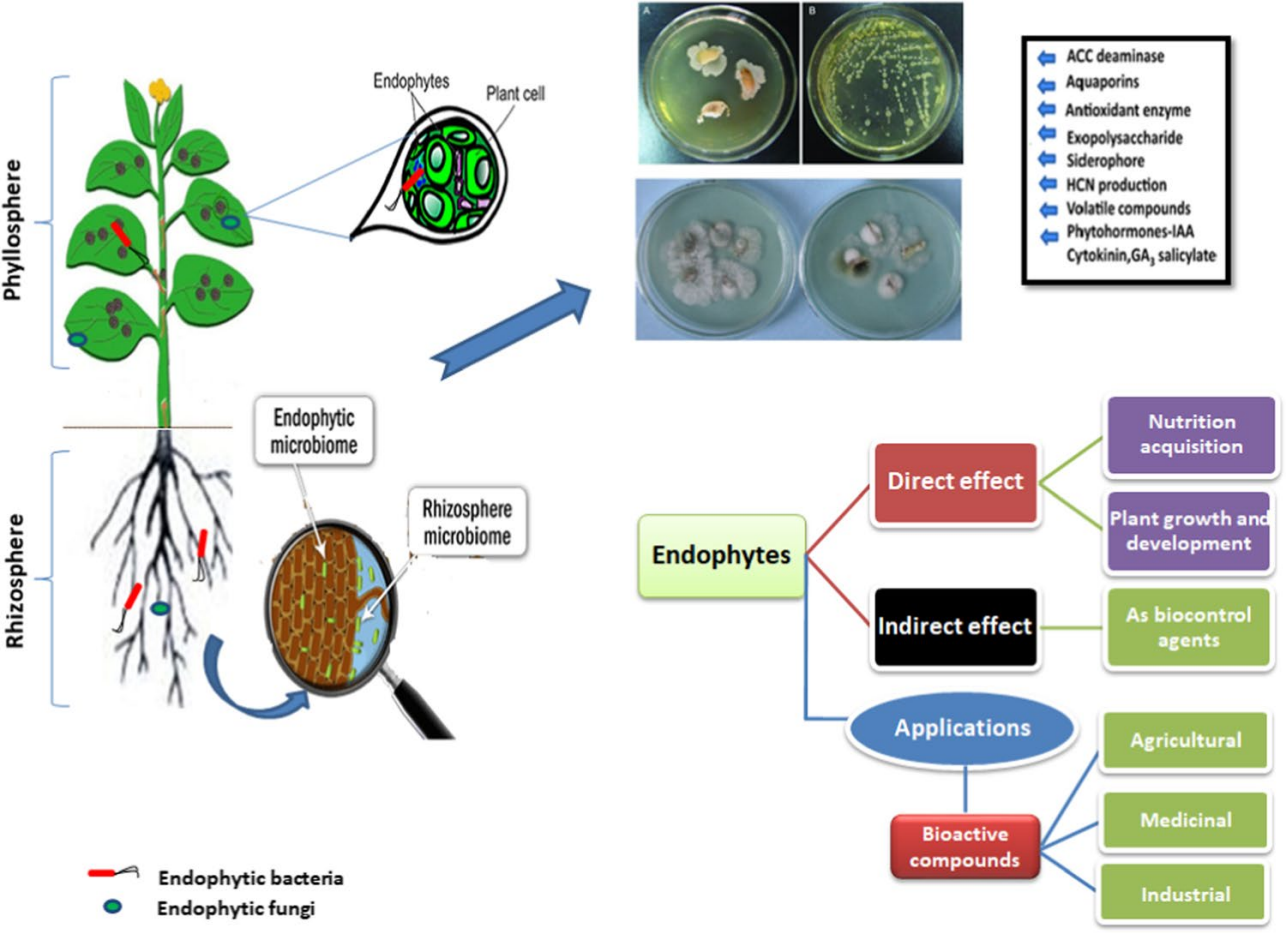
Overview of the plant–microbe interactions at phyllospheric and rhizospheric zone: endophytic microbes and rhizospheric microbes are capable to induce growth of the plants directly by increasing macronutrient and mineral uptake or indirectly through plant protection against pathogens. Naturally synthesized bioactive compounds with antimicrobial activities can be exploited in various sectors, especially in the agricultural and medicinal sectors

Plants interact with diverse microbial species thriving in the rhizosphere and phyllosphere, thereby resulting in altered vital biological activities together with defense strategies against various abiotic and biotic stresses [43, 78, 101, 178]. Rhizosphere and phyllosphere plant growth–promoting bacteria (PGPB) and mycorrhizal fungi in the rhizosphere are capable to induce growth of the plants directly by increasing macronutrient and mineral uptake and concentrations of essential hormones and/or indirectly through minimizing the negative impacts of a myriad of pathogens [18, 35, 79, 147, 161, 162, 171, 186, 240, 263] (Fig. 1).

The microbial species surviving on plant surfaces are epiphytes, whereas endophytes are those that inhabit the plant tissues [149, 203, 253]. In 1866, De Barry introduced the term “endophyte” for those organisms, including bacteria, fungi, or their associations multiplying intracellularly or intercellularly into host plants at least once in a lifetime without producing any marked signs of disease. Recent studies have illustrated that the growth and development of host plants depend to a greater extent on such symbiotic microbial species [55]. For example, in the most widely studied endosymbiotic association of rhizobium and legume, the bacterial counterpart is reported to regulate and meet the host plant nitrogen requirement [200, 201].

Endophytes facilitate the successful establishment of symbiotic association via the synthesis and secretion of plant growth–promoting compounds responsible for host adaptation under given environmental conditions. Several fungal, bacterial, and actinomycetes species are described to participate in the synthesis and secretion of biologically active compounds and secondary metabolites [7, 14, 46, 56, 64, 144, 189, 198, 230, 273].

Biomolecules belonging to classes of alkaloids, phenols, peptides, etc. synthesized by bacterial endosymbionts show a promising future in agriculture and medicine [163, 215]. For example, microbially synthesized bio-insecticide azadirachtin was found to be an effective inhibitor toward the desert locust (*Schistocerca gregaria*) [33]. Since its first discovery, azadirachtin has been found to be effective against more than 200 insect species and has become an active component of many commercial pesticides, including TreeAzin, AzaMax, BioNEEM, AzaGuard, and AzaSol [38, 59, 62, 80, 85, 94, 156, 196]. Many experimental investigations have reported the differential impact of factors such as specific host tissue, climatic conditions, and soil characteristics on bioactive compounds synthesized by endophytic microbial species [205]. The clue about the important role of endophytic microorganisms in the governance of the composition of metabolic products of host plants has attracted plant biologists to decipher the complexities of endophytic associations to improve crop plants.

**Fig. 2:**
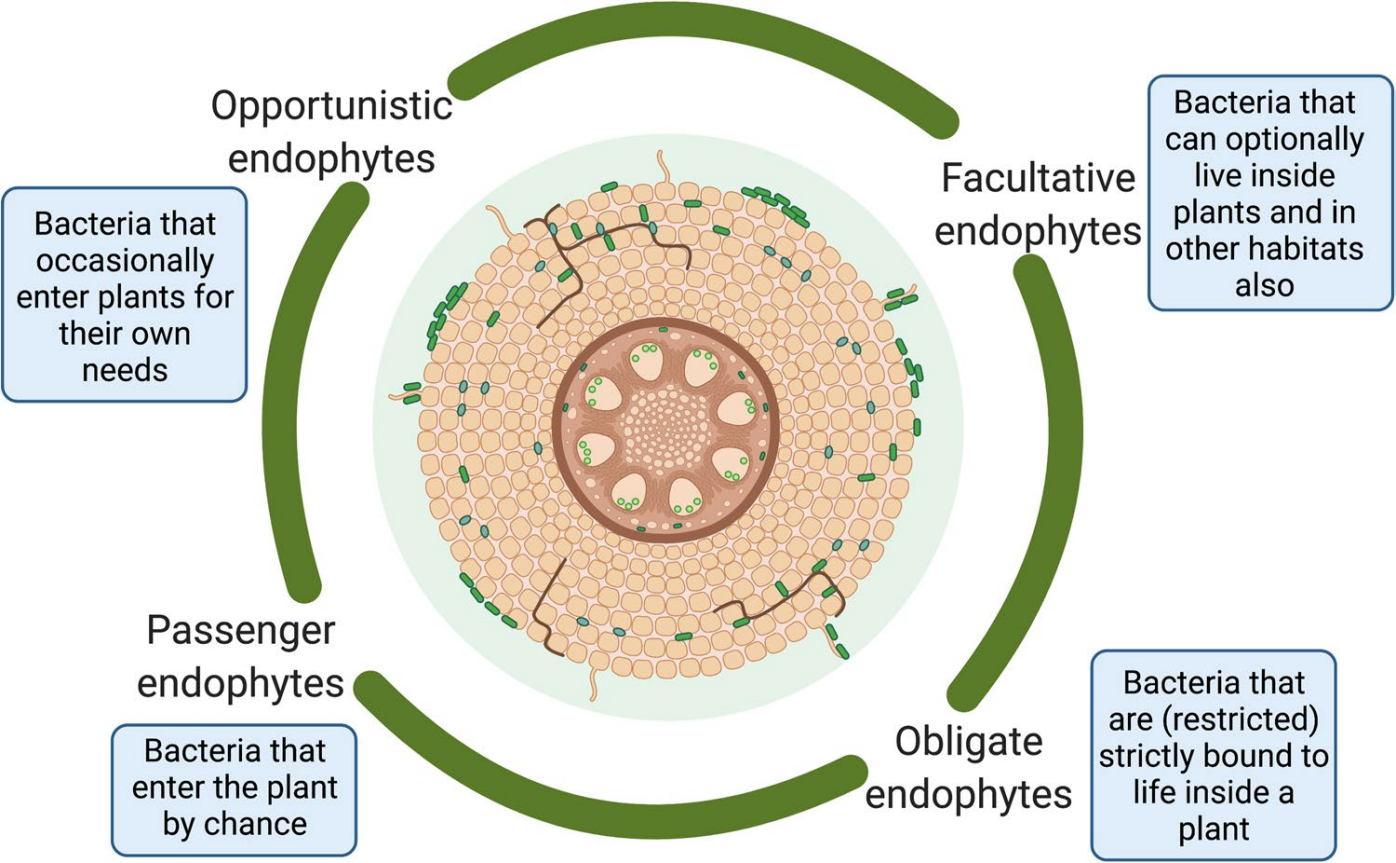
Categorization of endophytic bacteria based on their lifestyle. Opportunistic endophytes: they are bacteria which occasionally enter plants for their own needs. Passenger endophytes: they are bacteria which enter the plant by chance. Obligate endophytes: they are bacteria which are strictly bound to life inside a plant. Facultative endophytes: they are bacteria which can live inside plants and in other habitats also

Based on life strategies, endophytic bacteria were classified as facultative, obligate, opportunistic, and passenger endophytes [84] (Fig. 2). Currently, different biotic factors (e.g., insects and phytopathogens) and abiotic stress (e.g., extreme temperatures, salinity, drought, flood, low/excess nutrients, and organic/inorganic contamination) resulting from climate change have emerged as important limiting factors for agricultural and horticultural crop productivity worldwide [274]. Biotic stress has been estimated to reduce annual production of about 30% of crops [66]. In particular, combined effects of multiple abiotic stress factors such as drought and heat in a particular stage of growth of the plant are more detrimental than individual stress factors. Apart from abiotic stress factors, plants are constantly challenged with biological stresses through pathogenic bacteria, viruses, fungi, insects, and pests, causing considerable losses in food productivity worldwide [76, 152, 202, 234]. Various approaches, such as the selection of tolerant varieties, molecular breeding, and genetic engineering are being used to improve crop varieties against different stressors. However, the majority of these methods are time consuming, costly, and not well accepted in some areas [12]. Therefore, to neutralize the negative consequences of various factors connected with abiotic and biotic stress, host plants have developed many biological mechanisms that can function simultaneously. In this context, the mutualistic association arising from interconnections between the host and the microbe is considered an effective and sustainable means of improving plant development and growth [54, 132, 173, 195].

Unlike other plant growth–promoting microorganisms, endophytes have a direct relation with plants. They possess rapid adaptability under given conditions of biotic and abiotic stress, thereby improving host plant growth and survivability [9, 25, 61, 101, 149]. Furthermore, endophytic microbes can be an integral part of the rhizospheric region with the potential to synthesize and secrete metabolic products and enzymes [27, 188]. They facilitate in neutralizing harmful impacts of plant pathogens. They may also allow the host plant to multiply even in polluted soil by degradation of contaminants in a manner similar to those harbored by plant growth–promoting rhizobacteria (PGPR) [31, 37]. The application of high-throughput current “omics”-based technology such as gene sequencing, metabolomics, and microarray could comprehend the complex associations existing between plants and their endophytes and can be a promising tool for sustainable environmental development [40, 105]. Their high colonization efficacy and stability against abiotic stress make them a potential candidate for environmental management [12, 47, 116, 128].

The novelty of the present review is the current understanding pertaining to the colonization strategy of endophytes into host plants and their promising role in the alleviation of multiple abiotic and biotic environmental constraints limiting crop productivity. Noteworthy, the review has included comprehensive bibliometric information using the “SCOPUS” research database to illustrate the current research trend in the area of endophyte and possible implications in environmental stress management. In addition, the extensive information dealing with the possible roles of endophytes in eco-friendly removal of contaminants of hazardous nature including heavy metals, and diverse organic pollutants along with the future opportunities of endophytic microbes in crop improvement under changing climatic conditions, not considered in previously published reviews, are extensively taken into account.

## Study Design

This review was designed after a literature search and analysis using the following criteria to provide a critical, effective, and comprehensive analysis of the literature on endophytic microbes. A search was carried out with the SCOPUS database considering titles, abstracts, and keywords fields of all available literature. The search contained only two keywords: “endophytic” (or “endophyte”) and “stress.” It showed the publication of 2949 papers starting from 1960. To highlight the more recent results, the review was specifically addressed to the publications of the last 10 years (from 2012 until December 3, 2022), resulting in a total of 2532 publications.

To obtain a suitable and systematic synthesis of all bibliographic information, including the article title, abstract, authors, and keywords, a cluster analysis was performed using VOSviewer software (“VOSviewer version 1.6.16,” 2020).

**Fig. 3:**
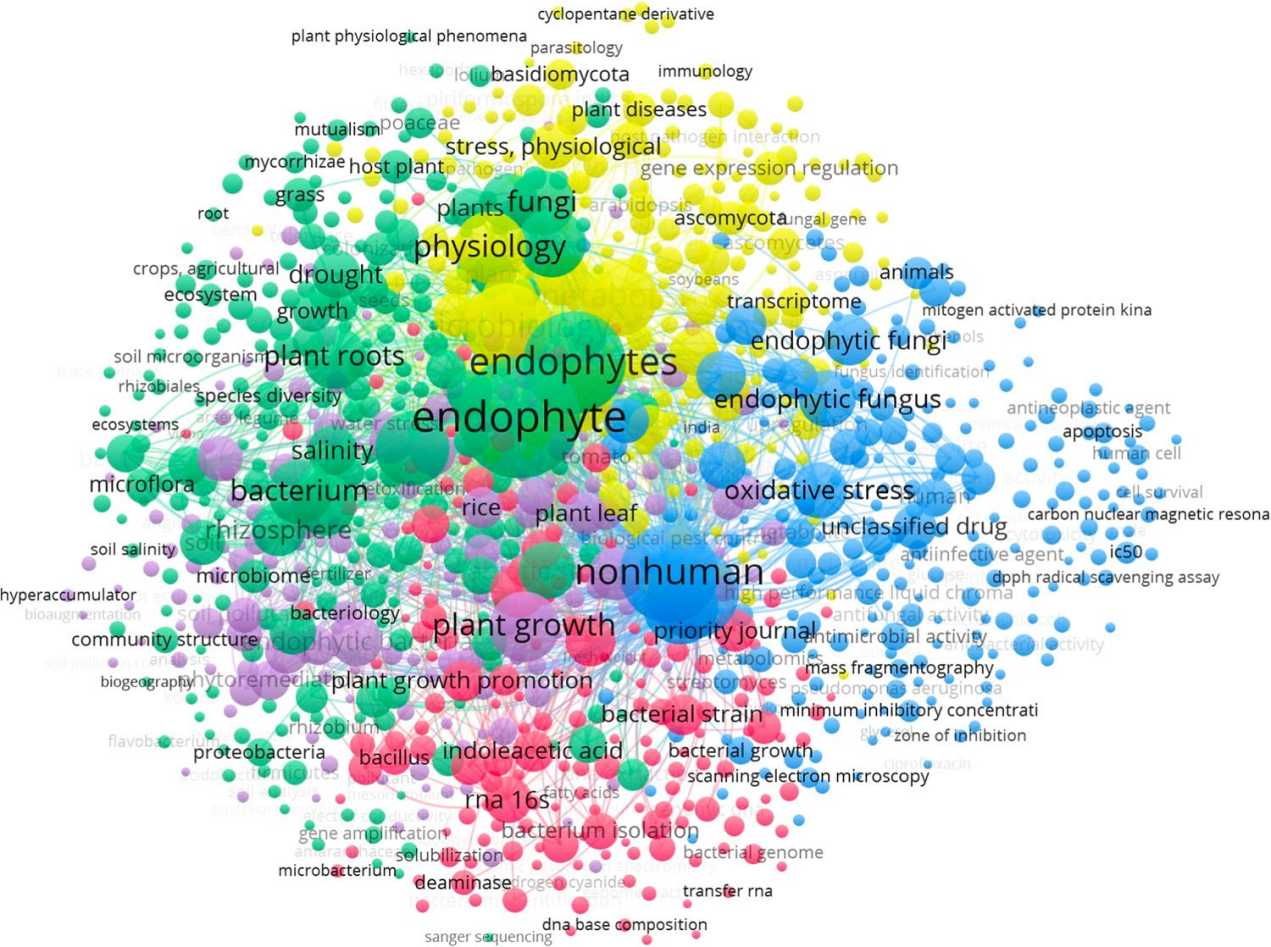
The keywords co-occurrence network, obtained from the articles extracted by SCOPUS, selecting two keywords: “endophytic” (or "endophyte") and “stress,” in the titles, abstracts, and keywords fields of all available literature. The review was specifically addressed to the publications of the last 10 years (from 2012 until December 3, 2022). The map highlights the most frequently used bibliographic terms to understand the most active research fields that are grouped into 5 clusters. Data analysis was performed by “VOSviewer version 1.6.16,” 2020

Figure 3 reports the cluster analysis provided from the co-occurrence network of keywords of the papers extracted from the SCOPUS platform. The results can be grouped into five clusters. The first cluster (241 items), highlighted by green balls, is devoted to stress factors and adaptation. Keywords are related to abiotic stress (e.g., salinity and drought) and biotic (pathogen).

Cluster 2 (224 items) keywords highlight the reactivity of endophytes, the endophytic production of metabolites, and the antibacterial activity of the obtained bioactive compounds (blue balls highlight this cluster). The third cluster is represented by yellow balls (192 items), and mainly concerns colonization mechanisms, with several keywords devoted to culture, and bacterial and fungi growth.

Cluster 4 (175 items), represented by violet balls, is devoted to remediation, with keywords related to contamination and detoxification. The keywords contaminants refer to heavy metals and organic pollutants. Finally, cluster 5 (168 items), represented by red balls, is mainly devoted to genome and genetic expressions.

Based on the study design, the review was conceived in the following sections:

## Impact of Stress Conditions on the Plant

The green revolution remarkably improved food availability in developing and developed countries. However, the indiscriminate use of chemical fertilizers reduced the biodiversity of soil microorganisms and frequently resulted in the loss of beneficial microbes necessary for soil health [126, 138]. Meanwhile, the predicted expansion of the human population beyond 10 billion in the next half a century requires doubling food production [232]. Therefore, ensuring stable global food production and supply is among the main challenges of the twenty-first century.

The biotic and abiotic stresses have a negative impact on agricultural productivity. Biotic stress includes pathogens that cause plant diseases (e.g., fungi, bacteria, viruses, and nematodes) and insects that feed on plant parts and compete with plants to get nutrients [178]. Phytopathogens can cause various plant diseases such as leaf spot, necrosis, wilt, head rot, fruit rot, root rot, and black foot [122, 146, 165]. In addition, insect feeding can cause bore formation on leaves, stems, flowers, and bark. Some insects are also potential vectors of microbial pathogens, so the disease becomes epiphytotic to healthy plant populations.

The main abiotic stress includes salinity, drought, nutrient-deficient, temperature (low/high), flood, heavy metal contamination stress, and ultraviolet radiation, which massively limit the overall yield and growth of crop plants [86, 231, 238, 246, 264, 271]. Drought stress alters the diffusion of nutrients, and the relationship between plants and water, and hampers normal functions, altering the plant morphological and physiological features. For example, drought stress can decrease chlorophyll content and cause an excess of reactive oxygen species (ROS) that can damage nucleic acids, proteins, and lipids [3, 41, 241, 255, 256]. Furthermore, salinity stress reduces the growth of plants and productivity through specific ion toxicity and osmotic effects that lead to nutritional imbalance, changes in morphology and biochemistry, and a decrease in photosynthesis [102, 137, 214]. In addition, the acidic condition can produce a nutrient deficiency in plants, leading to an acute loss of the physiological growth and development sequence. Heavy metals have a similar effect on plants,they are released into the soil, water, and atmosphere as a result of various anthropogenic activities such as industrialization, mining, and agricultural activities such as the use of fungicides, pesticides, and fertilizers, including organic ones. The concentration of heavy metals in the environment depends on different activities, then it can become toxic when it exceeds acceptable limits [199]. Finally, high- and low-temperature stress diminishes enzyme functioning, cell division, and excessive denaturation of membranous proteins that leads to cell death when the condition persists in the case of long-term conditions [28, 169]. Therefore, researchers need to develop sustainable microbe-based strategies to cope with difficult stress situations for food security and crop productivity. In this regard, endophytic microorganisms are the alternative that can contribute to plant health, nutrient supply, soil productivity, and protection against biotic and abiotic stress [49, 174, 176, 183].

## Colonization of Endophytes in the Host Plant

**Fig. 4:**
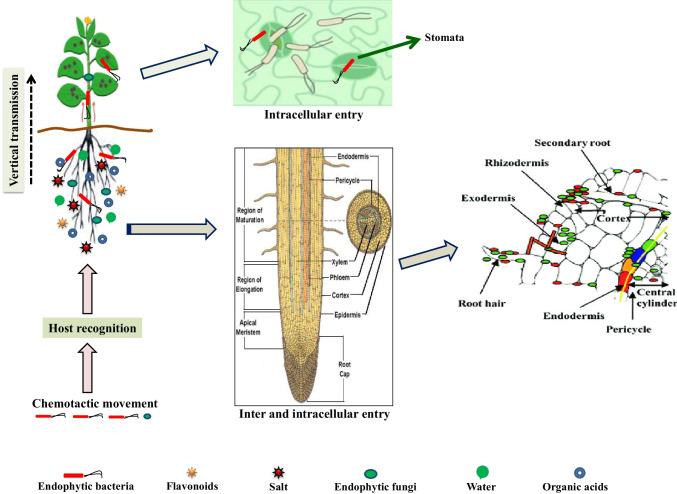
Entry and colonization of endophytic microorganisms in host plants. The successful colonization of the host plant by endophytes is a crucial component of advantageous plant–microbe interactions. Entry and colonization of endophytes into the host plant include several events that occur within the host plant, including endophytic population entrance, motility, transmission, and multiplication

Plant endophyte colonization cannot be considered an abrupt phenomenon, but a series of complex and organized events determined by chemotactic responses. The intracellular colony development mechanism adopted by bacteria and fungi is almost the same, but their strategies and modes differ considerably. For example, bacterial endophytes colonize intercellularly the host plant system vasculature, whereas fungal endophytes colonize inter- and intracellularly within the entire root system [103, 114, 129, 149, 179, 259]. The entry and colonization of endophytes involve different mechanisms comprising of (1) host availability and identification through a receptor and specific plant protein interaction, and (2) interaction with the phyllosphere followed by entry into the cellular environment (Fig. 4). Successful colonization by microbial endophytes is influenced by various factors such as the host plant genotype, the type of plant tissue, the microbial taxon and species, as well as abiotic and biotic stresses [135, 136]. Plant root exudates serve as chemical signals to attract bacterial endophytes. Bacteria use flagella to move toward the root surface and eventually leading to interaction with the plant system through pili and fibers [34, 113, 153]. During the moving process from the rhizosphere environment to the endosphere region, microbial endophytes can rapidly adapt to the contrasting environment (e.g., redox status, oxygen availability, nutrient composition, and the osmotic balance of the host cell system). Furthermore, microbial endophytes invading the endosphere region must cope with the host’s antioxidant defense machinery to internalize and colonize successfully [32, 113, 170]. In conclusion, the successful endophyte invasion and colonization within the host plant are largely determined by the timely identification of signaling substances, quorum responses, the potential to invade host defense machinery, and, most strikingly, the efficiency of tuning up with the entirely different complex host cellular system [119, 153].

## Role of Endophytes in the Management of Abiotic Stress

### Endophytes and Their Role in Mitigation of Drought and Temperature Stress

**Fig. 5:**
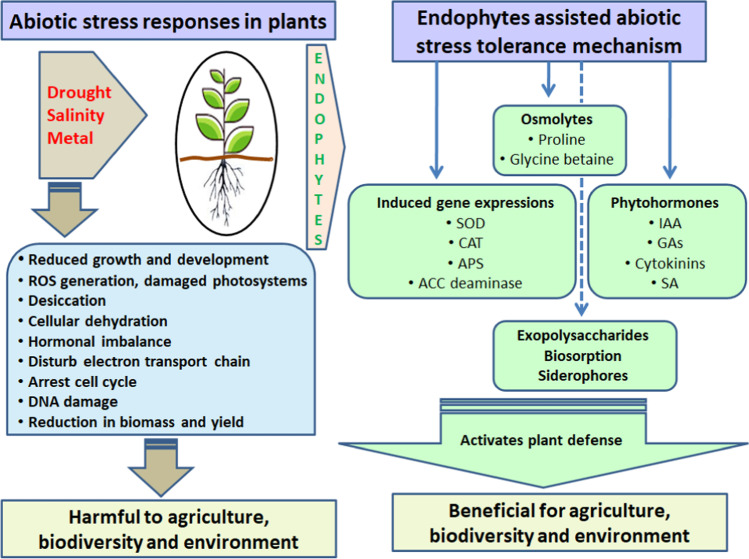
An overview of plant response to abiotic stress (left): prolonged abiotic stress (drought, salinity, and heavy metals) causes regeneration of ROS, desiccation, cellular dehydration, hormonal imbalance etc. that limit plant growth and productivity. Endophytic mediated abiotic stress tolerance mechanism (right): under abiotic conditions, endophytes trigger the production of osmolytes (proline, glycine betaine, etc.), secretion of phytohormones (IAA, cytokinins, GAs), and induce gene expressions for plant defense

Plants in natural environments are bound to expose to different abiotic stresses. Drought is one of the main limiting factors for the growth and productivity of crops around the world [58, 67, 177, 231]. Under water-limiting conditions, crop growth and productivity in the early stages are arrested due to low energy supply, low water uptake, and hindered functions of enzymes [52, 60, 121]. Furthermore, all considerable characters of plant–water relations, such as leaf relative water content (RWC), phenology, osmotic potential, water potential, pressure potential, photosynthesis, respiration, nutrition uptake, and rate of transpiration, are significantly impacted by drought, leading to decreased crop productivity (Fig. 5) [69, 83, 229]. Considerable research has been conducted for the development of resistance in various model and crop plant species using conventional and molecular techniques that are tedious and expensive. Therefore, researchers are seeking a sustainable approach and numerous studies recognize that plant-associated microbes have tremendous potential to develop resistance against drought.

The literature so far revealed that endophytes induce tolerance to drought by certain molecular and biochemical changes in plants [70, 208, 265, 269]. In field tests, the *Bukholderia phytofirmans* PsJN bacteria endophyte was inoculated in wheat plants that maintained metabolic balance due to higher antioxidant activity compared to control under drought conditions (Table 1) [164]. Furthermore, inoculation of the *Piriformospora indica* fungal endophyte also demonstrates drought resistance by upregulating antioxidant enzymes, drought-regulated genes, and CAS mRNA levels in drought-challenged leaves [223]. The pot experiment conducted on rice inoculated with *Trichoderma harzianum* TH-56 showed better drought tolerance by modulating SOD, proline, lipid peroxidation, and growth attributes, and the level of DHN/AQU transcript, under drought stress [181].

**Table 1:** An overview of endophytes mediating drought and salt tolerance and their physiological attributes in host plants

Abiotic stress	Site	Host plant	Isolated plant parts	Endophytes	Physiological responses in plants	References
Drought	Field trial; Experimental Farm of Institute of Soil and Environmental Sciences, University of Agriculture (UAF), Faisalabad	*Triticum aestivum* L. (Poaceae)	Roots	*Burkholderia phytofirmans* PsJN	Inoculation of *B. phytofirmans* PsJN improved the photosynthetic rate, water use efficiency and chlorophyll content	[164]
	Lab study; Zhejiang University, Huajiachi Campus, China	*Brassica rapa* L. (= *B. campestris* subsp. *chinensis* (L.) Makino) (Brassicaceae)	Root	*Piriformospora indica*	Inoculation of *P. indica* increased level of peroxidases, catalases, and superoxide dismutases, thus, inhibiting drought-induced degradation of chlorophyll and thylakoids proteins	[223]
	Pot experiment; G. B. Pant University of Agriculture and Technology, India	*Oryza sativa* L. (Poaceae)	Root	*Trichoderma harzianum* TH-56	Inoculation with increasing dose of *T. harzianum* strain Th-56 caused upregulation of aquaporin, dehydrin, and malondialdehyde genes	[181]
	Agriculture and Agri-Food Canada Research Centre, Canada	*Brachypodium distachyon* (L.) P. Beauv. (Poaceae)	Leaves	*Bacillus subtilis* B26	Endophyte-mediated up-regulation of *DREB2B-like*, *DHN3-like* and *LEA-14-A-like* and modulation of DNA methylation genes, *MET1B-like*, *CMT3-like* and *DRM2-like* genes that induce biochemical changes to overcome stress condition	[71]
	Agriculture and Agri-Food Canada Research Centre, Canada	*Phleum pratense* L. (Poaceae)	Leaves	*B. subtilis* B26	*B. subtilis* B26 modified osmolyte accumulation in roots and shoots	[70]
	Esmeraldas Province, Ecuador	*Theobroma cacao* L. (Malvaceae)	Pod	*Trichoderma hamatum* DIS 219b	Bacterial colonization caused drought-induced changes in stomatal conductance, net photosynthesis, and green fluorescence emissions	[21]
	Lab experiment; Institute of Biological Process Research, Japan	*Kalmia latifolia* L. (Ericaceae)	NM	*Streptomyces padanus*	Inoculation of *S. padanus* induced accumulation and lignification in cell walls in sieve cells conferred tolerance to drought in *Kalmia latifolia*	[88]
	Greenhouse experiment; Campus of Laboratório de Biologia Molecular de Plantas, Brazil	*Saccharum officinarum* cv. SP70-1143	NM	*Gluconacetobacter diazotrophicus*	Sugar plants colonized with *G. diazotrophicus* cause gene expression in shoots, contributing to drought resistance	[237]
	Lab experiment conducted in Crop Stress Biology for Arid Areas and College of Life Sciences	*Arabidopsis* sp. (Brassicaceae) and wheat (*Triticum* sp., Poaceae)	Leaves	*Pantoea alhagi* LTYR-11ZT	Strain LTYR-11ZT increased the contents of soluble sugars, but decreased proline, MDA and chlorophyll contents	[36]
	Gansu Province, northwest China	*Ammopiptanthus mongolicus* (Fabaceae)	Roots of *Gymnocarpos przewalskii* Bunge ex Maxim. (Caryophyllaceae)	Dark septate endophyte (DSE)	DSE enhanced root biomass and branch growth that might allow desert species to adapt in arid condition	[124]
	Field trial experiment at Sumter County and Stimpson Wildlife Sanctuary of southern Clarke County, USA Greenhouse experiment; Malayer University, Iran	*Solanum lycopersicum* L. (Solanaceae) *Solanum lycopersicum* L	Upper root and lower stem of *Pyrrhopappus carolinianus* (Walter) DC. (Asteraceae) Upper root and lower stem of *Pyrrhopappus carolinianus* (Walter) DC. (Asteraceae)	*Ampelomyces* sp. *Ampelomyces* sp.	*Ampelomyces* sp. enhanced strong root and shoot system under drought conditions. The overall study speculated that the improved health of the plant is due to the synergistic effects Symbiotic association between plant and fungal colonization increase the drought tolerance through morphological changes and molecular expression	[19, 160]
Salinity	Pot experiment; Chinese Academy of Forestry, Beijing, China	*Populus* × *tomentosa* Carrière (Salicaceae)	Roots of *Suaeda maritima* subsp. *salsa* (L.) Soó (= *S. salsa* (L.) Pall.) (Amaranthaceae)	*Curvularia* sp.	The endophytic fungi induced the elevated synthesis of the antioxidant enzymes SOD and APX. The inoculated plant expressed a high level of chlorophyll and proline content	[180]
	Pot experiment; CIMAP, Lucknow, India	*Chlorophytum borivilianum* Santapau & RR Fern (Asparagaceae)	Root	*Brachybacterium paraconglomeratum*	Bacterial ACC deaminase leads to ethylene reduction and its negative impact on plant growth	[23]
	Pot experiment; Fayoum University, Fayoum, Egypt	*Carthamus tinctorius* L. (Asteraceae)	Root, stem, and leaf	*Bacillus cereus* and *B. aerius*	Production of ACC deaminase causes ethylene reduction, thus lowering the negative impact on plant growth	[193]
	Pot experiment	*Oryza sativa* L. cv. KDML105 (Poaceae)	Roots of *Rotheca serrata* (L.) Steane & Mabb. (= *Clerodendrum serratum* (L.) Moon (Lamiaceae)	*Streptomyces* sp. GMKU 336	Endophyte enhanced the growth of rice by ethylene reduction via ACC deaminase and further assists plants in scavenging ROS, balancing the ion content and osmotic pressure	[98]
	Experimental farms in Ibaraki Prefecture, Tsukuba, Japan	*Solanum lycopersicum* L	Interior tissues of organic carrot and turnip crops, respectively	*Pseudomonas* sp. OFT2 and OFT5	ACC expressing endophyte alleviated salinity stress by reducing stress ethylene	[254]
	Pot experiment; King Abdullah University of Science and Technology Campus, Saudi Arabia	*Arabidopsis thaliana* (L.) Heynh. (Brassicaceae)	Root	*P. pseudoalcaligenes*	*P. pseudoalcaligenes* modulates Na^+^ and K^+^ ions under salt expression thus balance ion homeostasis	[4]
	Greenhouse experiment; Shanghai Jiao Tong University, China	*Brassica rapa* L. (= *B. campestris* subsp. *chinensis* (L.) Makino)	Roots	*Piriformospora indica*	Inoculated plants expressed higher activities of antioxidant enzymes, higher expression of genes conferring salt tolerance	[107]
	Field trial; desert region of Jizan, Saudi Arabia	*Tribulus terrestris* L. (Zygophyllaceae), *Tetraena simplex* (L.) Beier & Thulin (= *Zygophyllum simplex* L., Zygophyllaceae), *Panicum turgidum* Forssk. (Poaceae) and *Euphorbia granulata* Forssk. (Euphorbiaceae)	Roots	Endophyte isolate	Inoculation of endophytes conferred salinity tolerance in *A. thaliana* due to altered transporter transcripts, could be caused by the downfall of Na^+^/K^+^ shoot ratios	[50]
	Pot experiment; J.N.U, New Delhi, India	*Oryza sativa* L	Root	*Piriformospora indica*	Down-regulation of PiHOG1 confer salinity tolerance	[100]
	Greenhouse experiment; College of Food and Agricultural Sciences, Saudi Arabia	*Cicer arietinum* L. (Fabaceae)	Roots of *Acacia gerrardii* Benth. (Fabaceae)	*Bacillus subtilis* (BERA 71)	Enhancement in plant biomass, photosynthetic pigments, enzymatic and non-enzymatic antioxidant activity coupled with reduced ROS production and lipid peroxidation	[2]
	Field trial experiment at Sumter County and Stimpson Wildlife Sanctuary of southern Clarke County, USA Lab experiment at Root and Soil Biology Laboratory of the Botany Department, Bharathiar University, India Lab experiment; Zhengzhou University; China	*Solanum lycopersicum* L *A. thaliana* *Arabidopsis thaliana*	*Acer negundo* L. (Sapindaceae) Roots of *Chrysanthemum indicum* Isolated from salt-tolerant *Kosteletzkya* sp.	*Penicillium chrysogenum* *Fusarium haematococcum* *Bacillus cereus* KP120	Inoculation with *P. chrysogenum* showed increased salt tolerance at 300 mM of concentration Inoculation of endophytic *F. haematococcum* could induce salinity tolerance through production of extracellular enzymes under abiotic stress Up-regulation of key genes involved in IAA synthase and ethylene signaling were observed in *B. cereus* KP120 inoculated *A. thaliana* under salt-stressed condition	[160, 192] [266]

The accumulation of total soluble sugars, glucose, fructose, and starch content during endophyte infection plays an important role in increasing the resistance and improving plant tolerance to drought stress. *Bacillus subtilis* B26 has been found to reduce the negative effects of drought stress, which was linked to an increased level of starch content and total soluble sugars in inoculated stressed *Brachypodium distachyon* [71] and in *Phleum pratense* grasses [70]. The inoculation of the *Bacillus subtilis* B26 endophytic bacterium with *Phleum pratense* was found to have a significant effect on metabolism of plants. For instance, higher levels of fructans and sucrose, and key amino acids such as glutamic acid, glutamine, and asparagine were found in the roots and shoots of plants colonized compared to non-colonized ones. Furthermore, inoculation of plants with endophytes resulted in an increased level of a non-protein amino acid, i.e., gamma-aminobutyric acid (GABA), in shoots and roots [70, 92]. A *Trichoderma hamatum* DIS 219b fungal endophyte delayed the onset of drought response in *Theobroma cacao* by changing gene expression, possibly corresponding to changes in net photosynthesis, stomatal conductance, and green fluorescence emissions [21]. A recent study indicated that *Ampelomyces* sp. colonized tomato plants and improved the promotion of plant growth under drought conditions, representing a sustainable form of biofertilizer that could improve agronomic production [160]. The recent finding revealed that *P. indica* confers drought tolerance by the regulation of promoter genes, resulting in morphophysiological changes in tomatoes [19]. In summary, the endophyte-mediated drought resistance mechanism is based on phytohormone production, antioxidant-mediated ROS scavenging activity, induction of microbial genes, and accumulation of compatible solutes (Fig. 5).

In turn, to alleviate heat/temperature stress (HS), some studies have identified the potential role of plant hormones and other secondary metabolites produced by fungi endophytes such as *Paecilomyces formosus* LWL1 in the *Dongjin japonica* rice cultivar. This fungus protected rice plants against HS compared to the control, as shown by lower endogenous stress signaling compounds, such as jasmonic acid (34.57%) and abscisic acid (25.71%), and the overall protein content increased (18.76–33.22%) [245]. The *Rhizopus oryzae* endophytic fungus inoculated in soybean (*Glycine max* L.) and sunflower (*Helianthus annuus* L.) also has the potential to alleviate thermal stress. Namely, both crops also showed low levels of abscisic acid (ABA), while high levels of catalase (CAT), ascorbic acid oxidase (AAO), phenolics, proline, sugars, flavonoids, lipids, and proteins were also observed. It was also found that the endophytic fungus stimulates chlorophyll content, length of shoots and roots, and dry and fresh biomass compared to uninoculated plants [97]. *Aspergillus japonicus* EuR-26 endophytic fungus isolated from the *Euphorbia indica* L. wild plant (*Euphorbiaceae*) also mediated the growth of host plants under normal and heat-stress conditions. Namely, *A. japonicus*–associated sunflower and soybean seedlings improved the growth of plant biomass and other plant traits and food quality (flavonoids, phenolic, proteins, soluble sugars, and lipids) under the stress of high temperature (40 °C) compared to plants without endophyte [96]. These types of phenomena are also observed in wild plants, e.g., in the desert plant *Cullen plicatum* (Delile) C.H.Stirt. (*Fabaceae*) which, if it is a co-inhabitant with another endophytic fungus, *Thermomyces lanuginosus*, copes much better with heat stress in its natural environment [11].

### Endophytic Microorganisms and Their Role in Alleviating Salinity Stress

Salinity is one of the most important environmental problems affecting plant productivity in dry and semi-dry climates [6, 216, 102, 133, 260]. The high salt content of the soil has been described as the result of natural and human activities leading to soil sodium salt accumulation. Furthermore, soil high salt concentration is frequently correlated with the reduction in seedling formation and imbalance in cellular homeostasis culminating in diminished photosynthetic activities [13, 204, 222, 267].

Endophytic microorganisms develop strategies against salinity, similar to drought-resistant mechanisms. Endophytes stimulate the synthesis of antioxidant enzymes to balance various free radicals and maintain the normal functioning of the cell under salinity stress (Table 1). For example, inoculation of poplar tree with *Curvularia* sp. stimulates plant production of ascorbate peroxidase (APX) and superoxide dismutase (SOD) [180]. Furthermore, exposure of endophytic microbes to high salinity may stimulate the synthesis of the ACC deaminase. For instance, Barnawal et al. [23] observed an increase in the growth rate of salt-sensitive spider plants (*Chlorophytum* sp., *Asparagaceae*) with the presence of the bacterium *Brachybacterium paraconglomeratum* that produce ACC deaminase and diminishing the negative impact of gaseous hormone ethylene. Similar studies on the involvement of ACC deaminase for improved rice plant growth and stress mitigation were recently described [98, 193, 254].

In addition, osmolyte production was also recorded in maintaining the sodium–potassium ratio to overcome the osmotic effect of salinity (Table 1).

The pot experiment demonstrated that colonization with *P. pseudoalcaligenes* improved *Arabidopsis* sp. growth under salt stress conditions by likely modulating the expression levels of K^+^ and Na^+^ ion channels and genes involved in Na^+^/K^+^ homeostasiss [4]. Colonization of *P. indica* in salinity-sensitive *Brassica rapa* (= *B. campestris* subsp. *chinensis*) confers salinity tolerance by significantly higher production of antioxidant enzymes such as catalase (CAT), peroxidase (POD), and SOD and increased the plant hormone level such as gibberellic acid (GA) and salicylic acid (SA) [107]. Further study by Ravi et al. (2022) suggested that fungal root endophyte (*Fusarium haematococcum*) can resist salt stress and produces extracellular enzymes such as amylase, cellulase, and protease under in vitro conditions in addition to antioxidant production [192].

Recently, Eida et al. [50] have illustrated the role of endophytes isolated from desert plants in mitigating plant stress caused in the soil by the high salt content. The model plant *Arabidopsis thaliana* exposed to different salt levels exhibited tolerance to salinity after inoculation of isolated endophytes. Recent findings of Zhang et al. [266] concluded that apart from higher antioxidative enzymes of proline content, upregulation of key genes involved in IAA synthase and ethylene signaling were observed in *B. cereus* KP120 inoculated with *A. thaliana* under salt-stressed condition. In addition, a number of recent research have shown that isolated endophytes are very effective in enhancing physiological performance, plant growth, root and shoot biomass, symbiotic performance, energy production, osmoregulation, Na^+^ sequestration, and ion homeostasis under salt-stressed conditions [30, 48, 110, 111, 123, 125, 157, 160, 219].

## Role of Endophytes in the Management of Biotic Stress

**Fig. 6:**
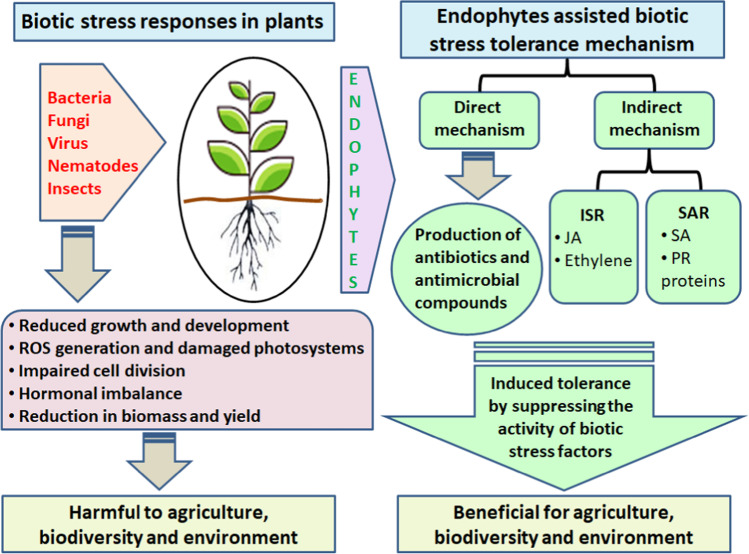
An overview of plant response to biotic stress (left): pathogen infection causes photosystem damage, ROS regeneration, and impaired cell division that lead to reduced plant growth and development. Endophytic mediated biotic stress tolerance mechanism (right): endophytes trigger defense mechanisms directly by the production of antimicrobial compounds and indirectly through the production of lytic enzymes, activation of systemic defense responses involving jasmonic acid (JA), oligogalacturonoids (OGAs), and salicylic acid (SA) signaling pathways

Plants are often exposed to harmful molecules produced by microorganisms. These molecules alter plant metabolism, causing diseases and significant crop loss [53, 76, 217]. Beneficial interactions between plants and microbes play an important role in plant protection against phytopathogens. Plant-beneficial microorganisms release elicitors that alter biochemical and physiological plant properties in changing environments [5, 34, 99]. Plants have physical and chemical barriers able to react to pathogens: they activate signal transduction in response to pathogen attacks directed to induce defenses. Important mechanisms of tolerance to biotic and abiotic stress are ROS production, antioxidative defense, and oxidative burst [72, 87, 151, 218, 261]. Like rhizosphere microbes, endophytes trigger direct and indirect mechanisms of disease resistance (Fig. 6). Direct mechanisms include the production of antimicrobial compounds and the lytic enzymes of the cell wall of fungi are capable of inhibiting plant pathogen growth and act as biological controls (Table 2). For example, a study suggested that chitinase produced by endophytic *Streptomyces* sp. can control plant pathogenic fungi [187].

**Table 2:** Summary of endophyte metabolites used in biological control of phytopathogens and plant diseases

Site	Plant parts	Endophytes	Metabolites	Diseases/pathogens	References
Lytic enzymes					
Lab condition; Microbial Department of Genetics, Piracicaba, Brazil	Tissue of *Citrus* sp. (Rutaceae) and *Glycine max* (L.) Merr. (Fabaceae)	*Streptomyces* sp.	Chitinase	*Colletotrichum sublineolum*	[187]
Lab experiment; Jiangsu Key Laboratory for Microbes and Functional Genomics, Jiangsu Province, Nanjing, China	Leaves of *Atractylodes lancea* (Thunb.) DC. (Asteraceae)	*Pseudomonas fluorescens*	Amylase, xylanase, cellulose, and pectinase	*Athelia rolfsii/Blast*	[268]
Antimicrobial compounds					
Lab experiment; Jinju area, Korea	Root of *Artemisia* sp. (Asteraceae)	Pseudomonads	DAPG	*Verticillium dahliae*, *Colletotrichum gloeosporioides*, *Fusarium oxysporum*, and *Phytophthora capsici*	[39]
Lab experiment; Garut, West Java Indonesia	Stem of potato	*Paracoccus halophilus* G062	DAPG and pyrrolnitrin	Inhibit the growth of pathogens	[10]
Lab experiment; Forks Natural Area, USA	Root and stem of poplar and willow	*Burkholderia* sp., *Rahnella* sp., *Pseudomonas* sp., and *Curtobacterium* sp.	Occidiofungin and hydrogen cyanide	*Rhizoctonia solani* AG-8, *Fusarium culmorum*, *Gaeumannomyces graminis* var. *tritici* and *Pythium ultimum*	[103]
		*Paraconiothyrium* SSM001		*Heterobasidion annosum*, *Phaeolus schweinitzii*, and *Perenniporia subacida*	[220]
	Leaves	*Phomopis cassiae*	3,11,12-Trihydroxycadalene	*Cladosporium sphaerospermum* and *Cladosporium cladosporioides*	[213]
Lab experiment; Dong Zai, Hainan, China	Stem of *Excoecaria agallocha* L. (Euphorbiaceae)	*Phomopsis* sp.	Phomopsins A, B, C, Cytosporone	*Candida albicans* and *Fusarium oxysporum*	[93]
Lab experiment; Jiangsu Key Laboratory for Microbes and Functional Genomics, Jiangsu Province, Nanjing, China	Leaves of *Atractylodes lancea* (Thunb.) DC	*Pseudomonas fluorescens*	2-Piperidinone, DAPG, siderophore	*Athelia rolfsii*/Blight	[268]
		Endophytic bacteria	Siderophore	*Fusarium oxysporum*	[182]
Shell Islands of the Yellow River Delta, China	*Flueggea suffruticosa* (Pall.) Baill. (= *Securinega suffruticosa* (Pall.) Rehder, Phyllanthaceae)	Endophytic fungi	Antifungal compounds		[45]
Field trial; WSU Mount Vernon, Northwestern Washington Research and Extension Center, USA	Roots of *Cucurbita pepo* L. (Cucurbitaceae)	*Dichotomopilus/Chaetomium* sp., *Cladosporium* sp., *Clonostachys* sp., *Epicoccum* sp., and *Fusarium* sp.	Antifungal compounds	*Verticillium dahliae*	[235]
PR proteins					
Lab experiment; British Columbia, Canada	Roots of Chinese cabbage	*Heteroconium chaetospira*	PR2 (β-1,3-glucanase)	Club rot; *Plasmodiophora brassicae*	[120]
Lab experiment; Institute of Environmental Biotechnology, Greece	Root tissue of tomato	*Fusarium solani*	PR5 and PR7	*Fusarium oxysporum*	[106]
Lab experiment; Research Center for BioSystems, Justus Liebig University, Giessen, Germany	Roots	*Piriformospora indica*	PR1, PR2, and PR5	Powdery mildew; *Blumeria graminis* f. sp. *hordei*	[158]

Many fungal and bacterial endophytes produce antimicrobial compounds with strong antifungal and antibacterial activities that could be antagonistic to plant pathogens [7, 51, 108, 141, 142, 145, 155, 228]. For example, endophytes *Pseudomonas* sp. isolated from *Artemisia* sp. roots (Asteraceae) known to produce the antibiotic DAPG (2,4-diacetylphloroglucinol) can also induce the defense of plants against pathogens such as *Verticillium dahliae*, *Colletotrichum gloeosporioides*, *Fusarium oxysporum*, and *Phytophthora capsici* (Table 2) [39]. In addition, the DAPG-producing bacterium *Paracoccus halophilus* G062 can aggressively colonize stems and leaves, and further suppress pathogen establishment [10]. *Populus trichocarpa* and *Salix sitchensis* (both Salicaceae) are dominant endophytes taxonomically affiliated with *Burkholderia*, *Rahnella*, *Pseudomonas*, and *Curtobacterium* genera. These genera are well known for producing antifungal compounds (e.g., occidiofungin and hydrogen cyanide) with proven biocontrol activities against soil-borne plant pathogens, including *Fusarium culmorum*, *Rhizoctonia solani* AG-8, *Pythium ultimum*, and *Gaeumannomyces graminis* var. *tritici* [103].

Like bacterial endophytes, it has been reported that fungal endophytes produce antimicrobial compounds. For instance, Soliman et al. [220] reported that the *Paraconiothyrium* endophyte strain SSM001 inhibits the growth of *Heterobasidion annosum*, *Phaeolus schweinitzii*, and *Perenniporia subacida* wood-decaying fungal species. Furthermore, the 3,11,12-trihydroxycadalene (sesquiterpenes derivatives) produced from the endophytic fungus *Phomopis cassiae* isolated from *Senna spectabilis* (DC.) H.S.Irwin & Barneby (= *Cassia spectabilis* DC., *Fabaceae*) has been reported as a strong antifungal agent against *Cladosporium cladosporioides* and *C. sphaerospermum* [213]. Similarly, *Flueggea suffruticosa* (= *Securinega suffruticosa*, *Phyllanthaceae*) and *Cucurbita pepo* (*Cucurbitaceae*) were colonized by fungal endophytic isolates that inhibited the growth of respective pathogens of plants [45, 235].

A variety of microbial phyla, including *Pseudomonas* sp., *Bacillus* sp., and *Trichoderma* sp., have been shown to lead to systemic resistance in plants against pathogen attacks [117, 150, 168, 175, 184]. Microorganisms activate defense reaction mechanisms that involve the induction of systemic acquired resistance (SAR) and systemic resistance (ISR) pathways. SAR is activated by pathogen infection, which is connected with the activation of salicylic acid signaling and the accumulation of pathogenesis-related proteins (PR). For example, activation of β-1,3-glucanase (PR 2) was increased in oilseed rape infected with *Plasmodiophora brassicae* after colonization with *Heteroconium chaetospira*, a dark septate endophyte [120]. Similarly, the endophyte *Fusarium solani*, recovered from tomato, triggered ISR across the *Septoria lycopersici* tomato foliar pathogen and activated the expressions of PR7 and PR5 in roots [106]. Experimental studies on resistance induction mediated by the endophyte *Serendipita indica* revealed that *Blumeria graminis* f. sp. *hordei* inoculation resulted in induction of gene expressions (notably Hsp70, PR1, PR2, and PR5, and barley chemically induced 7 (BCI-7)) in barley foliage, which is supposed to be involved in various functions including defense reactions and protein synthesis and apoptosis [158].

## Role of Endophytic Microorganisms in Phytoremediation

### Phytoremediation of Heavy Metals

Currently, the management of environmental pollutants based on living agents has achieved considerable progress worldwide. Pollutant removal by photosynthetic organisms (e.g., phytoremediation) has emerged as an attractive and light-driven decontamination technique and also an emerging green sustainable technology [44, 63, 65, 89, 118, 185, 221, 226, 233, 236]. However, the low multiplication rate along with the low amount of cell mass, phytotoxic impacts, and release of pollutants of gaseous nature are the main drawbacks associated with phytoremediation technology, making the process inefficient at field scale [26, 73, 242, 252]. The solution to these limitations lies in the development of microbe-assisted phytoremediation. Previous studies have illustrated the use of rhizosphere-dwelling microbes to improve pollutant removal [77, 81, 190, 250, 275]. Furthermore, it was suggested that endophytes could facilitate phytoremediation more efficiently [44, 112, 154].

The negative impacts of heavy metals on plants can be described as reduced crop productivity resulting from changes in growth rate, nutrient accumulation capacity, and leaf area. In addition, heavy metal pollutions can cause considerable changes in community structure of diverse microbial populations and function associated with host plants [29, 42, 224]. Numerous studies have discussed the impact of various heavy metals on the diversity of endophytes, biological processes, and biomass production [57, 68, 123, 125, 127, 172].

However, current studies dealing with the interactions between hyperaccumulator plants and endophytes have attracted attention worldwide because of inherent pollutant removal ability and possibilities for large-scale applications [91, 109, 123, 125, 130, 131, 197, 225, 243]. Furthermore, hyperaccumulators sequester a significantly high content of hazardous heavy metals and therefore create the internal environmental conditions suitable for the development of metal resistance in endophytes exposed to high heavy metal concentrations [172].

In terms of endophytic application, various metal-resistant endophytic bacteria were isolated from leaves, stem, and roots of plant hyperaccumulators, including *Thlaspi caerulescens*, *Th. goesingense*, *Alyssum bertolonii* (all *Brassicaceae*), and *Nicotiana tabacum* (*Solanaceae*) (Table 3). The association of these endophytes with hyperaccumulators suggests the widespread habitat choice of these microbes. For example, *Thlaspi goesingense* stems under field conditions harbor different bacteria including α-proteobacteria, γ-proteobacteria, *Acidobacterium* sp., *Bacillus* sp., *Blastococcus* sp., *Curtobacterium* sp., *Desulfitobacterium metallireductans*, *Flavobacterium* sp., *Holophaga* sp., *M. mesophilicum*, *M. extorquens*, *Plantibacter flavus*, *Propionibacterium acnes*, *Rhodococcus* sp., and *Sphingomonas* sp. These isolates were shown to be resistant to nickel (Ni) concentrations between 5 and 12 mM [95]. The same results were obtained in the field site experiment that the total Ni uptake by *Alyssum serpyllifolium* (*Brassicaceae*) was significantly enhanced by heavy metal–resistant endophytic bacterial strains *Microbacterium* sp., *Pseudomonas* sp., and *Staphylococcus* sp. [24]. In the line of the same experiment, Ma et al. [134] found that inoculation with the plant growth–promoting *Pseudomonas* sp. A3R3 endophytic bacterium significantly increased Ni uptake by 10% in *A. serpyllifolium*. In a later experiment, *Achromobacter piechaudii* was documented to sequester more than 60% of zinc (Zn), lead (Pb), and cadmium (Cd) from the corresponding hyperaccumulators, namely, *Sedum plumbizincicola* (*Crassulaceae*), *Alnus firma* (*Betulaceae*), and *Solanum nigrum* (*Solanaceae*), respectively [135, 136]. Similarly, another study reported arsenic (As)-tolerant *Bacillus* sp. endophytes isolated from the leaves, stem, and root of *Pteris vittata* and *P. multifida* (*Pteridaceae*) [270] and concluded that bacteria with less biomass had greater tolerance to As. Surprisingly, fungal endophytes *Fusarium* sp. CBRF44, *Alternaria* sp. CBSF68, and *Penicillium* sp. CBRF65 isolated from the hyperaccumulators *Brassica napus* (*Brassicaceae*) showed significant tolerance to Cd and Pb [211]. This finding supported the result of Zhu et al. [272] where dark septate endophytes *Phialophora mustea* inoculated tomato roots established remarkable tolerance to Cd and Zn and promoted the tomato seedlings’ growth under all metal stresses tested.

**Table 3:** An overview of endophytes involved in the phytoremediation of heavy metals from hyperaccumulation (H) and non-hyperaccumulation (NH) in plants

Site	Hyperaccumulators (H)/non-hyperaccumulators (NH)	Plant parts	Metal-resistant endophytes	Heavy metals	Heavy metal tolerance capacity	References
Field site; Redlschlag, Eastern Austria	*Thlaspi goesingense* Halácsy (Brassicaceae) (H)	Stems	α-Proteobacteria, β-proteobacteria, γ-proteobacteria, *Acidobacterium* sp., *Bacillus* sp., *Blastococcus* sp., *Curtobacterium* sp., *Desulfitobacterium metallireductans*, *Flavobacterium* sp., *Holophaga* sp., *M. mesophilicum*, *M. extorquens*, *Plantibacter flavus*, *Propionibacterium acnes*, *Rhodococcus* sp., and *Sphingomonas* sp.	Ni	Isolates were resistant to Ni concentrations between 5 and 12 mM; however, endophytes generally tolerated higher levels of Ni than rhizosphere bacteria	[95]
Field site; Galceti, a serpentine outcrop located in Tuscany (Italy)	*Alyssum bertolonii* Desv. (Brassicaceae) (H)	Leaves	*Microbacterium* sp., *Pseudomonas* sp., and *Staphylococcus* sp.	Ni	Most endophytes showed Ni tolerance up to 5 mm concentration	[24]
		Stem	*Curtobacterium* sp., *Microbacterium* sp., and *Staphylococcus* sp.			
		Root	*Arthrobacter* sp., *Bacillus* sp., *Curtobacterium* sp., *Leifsonia* sp., *Microbacterium* sp., *Paenibacillus* sp., *Pseudomonas* sp., and *Staphylococcus* sp.			
Field site; serpentine soils in Braganca, NE of Portugal	*Alyssum serpyllifolium* Desf. (Brassicaceae) (H)	NM	*Pseudomonas* sp. A3R3	Ni	A3R3 isolate inoculated *A. serpyllifolium* increased Ni uptake by 10% when plants were grown in soil amended with 450 mg Ni kg^−1^	[134]
Field site; mine soils in Chunan city of Zhejiang, Southeast of China	*Sedum plumbizincicola* X.H. Guo & S.B. Zhou (Crassulaceae) (H)	Roots, stems, and leaves	*Achromobacter piechaudii*	Cd, Zn, Pb	The highest metal biosorption content was observed with Zn (10.9 mg/g of dry cell weight), followed by Cd and Pb	[135, 136]
Greenhouse experiment; Nanjing University, Jiangsu, China	*Pteris vittata* L. and *Pteris multifida* Poir. (Pteridaceae) (H)	Root, stems, and leaves	*Bacillus* sp. isolates	As	All 42 isolates showed tolerance to As(V), while some tolerated As(III). Endophytes isolated from *Pteris vittata* were tolerant to As(V), while endophytes of *Pteris multifida* showed tolerance to As(III)	[270]
Field experiment; heavy metal–contaminated site; Guangdong Province, China	*Brassica napus* L. (Brassicaceae) (H)	Stems and roots	*Fusarium* sp. CBRF44, *Alternaria* sp. CBSF68, *Penicillium* sp. CBRF65	Pb, Cd	Endophytes showed a significant tolerance level; *Fusarium* sp. CBRF44 was resistant to 5 mM Cd and 15 mM Pb, *Alternaria* sp. CBSF68 was resistant to 1 mM Cd and 10 mM Pb; *Penicillium* sp. CBRF65 had a tolerance level of 2 mM Cd and 20 mM Pb	[211]
Field experiment; agricultural soil of Chenggong county, Yunnan Province, SW China	*Lycopersicon esculentum* Mill. (Solanaceae) (H)	Root	*Phialophora mustea* Neerg. (K36 and Z48)	Zn, Cd	Endophyte inoculated tomato plants showed a lower accumulation of Cd and Zn in both the shoots and roots, excluding slightly reduced shoot values for K36-inoculated treatments under 5 mg/kg Cd and 300 mg/kg Zn	[272]
Greenhouse experiment; Nanjing, East China	*Brassica napus* L. (H)	Roots	*Microbacterium* sp. G16 and *Pseudomonas fluorescens* G10	Pb	Pb-resistant strains *P. fluorescens* G10 and *Microbacterium* sp. G16 significantly increase total Pb uptake in shoots. Inoculation with G10 strains increased shoot total Pb uptake from 76 to 131% and from 59 to 80% (*p* < 0.05) for strain G16, respectively	[209, 210]
Metal-enriched sandy loamy soil near Zurich Airport, Switzerland	*Nicotiana tabacum* L. (Solanaceae) (H)	Seeds	*Clostridium aminovalericum*, *Enterobacter* sp., *Pseudomonas* sp., *P. fulva*, members of *Xanthomonadaceae*, *Sanguibacter* sp., and *Stenotrophomonas* sp.	Cd	Inoculation of Cd-resistant *Sanguibacter* sp. in *Nicotiana tabacum* increased the concentration of Cd in shoot tissues	[148]
Field site; Japanese National Forest, Hitachi mine, Ibaraki prefecture, Japan	*Clethra barbinervis* Siebold & Zucc. (Clethraceae) (H)	Root segment and leaves	*Phialocephala fortinii*, *Rhizodermea veluwensis*, and *Rhizoscyphus* sp.	Cu, Zn, Ni, and Pb	*C. barbinervis* can tolerate high concentrations of heavy metals (Cu, 2–1123 μg/g; Zn, 21–2600 μg/g; Pb, 32–1506 μg/g) due to the support of root fungal endophytes including *P. fortinii*, *R. veluwensis*, and *Rhizoscyphus* sp. via growth enhancement, K uptake promotion, and decrease of heavy metal concentrations	[257]
Field site; Pb-contaminated mining, Korea	*Alnus firma* Siebold & Zucc. (Betulaceae) (H)	Roots	*Bacillus* sp.	Pb, Cu	Two isolates designated MN1-5 and MN3-4 showed resistance to Pb up to 1500 mg/L concentration	[212]
Sewage discharge canal bank of Zhuzhou Smeltery, China	*Solanum nigrum* L. (Solanaceae) (H)	Roots, stems, and leaves	*Bacillus* sp.	Cu, Cd, Cr	At 10 mg/L of heavy metals (Cu, Cd, Cr), strain EB l4 potentially uptakes 75.78%, 80.48%, and 21.25% Cd(II), Pb(II) and Cu(II) within 24 h of incubation	[82]
Field site; sewage discharge canal bank of Zhuzhou, Smeltery, China	*Solanum nigrum* L. (H)	Roots, stems, and leaves	*Serratia* sp.	Cd	The endophytic bacterium LRE07 detoxifies 65% of cadmium	[130, 131]
Field experiment; mining site of Huize County, Yunnan Province, Southwest China	*Arabis alpina* L. (Brassicaceae) (H)	Roots, shoots, and seeds	*Tetracladium* was reported as the dominant fungal endophyte in roots and shoots, while *Alternaria* reported in seeds	Pb, Zn		[206]
Arnoldstein, Austria	*Salix caprea* L. (Salicaceae) (NH)	Stems and leaves	*Frigoribacterium* sp. and *Microbacterium* sp., *Methylobacterium* sp., and *Sphingomonas* sp.	Zn, Cd	Endophyte inoculation improved the accumulation of Zn, Cd in leaves; endophyte inoculated *Salix* sp. extracted in sterile TSB medium extracted 2.62 mg of Zn and 173 µg Cd per kg of soil	[115]
Greenhouse experiment Pb–Zn mining sites of Huize County, Yunnan Province, Southwest China	*Oryza sativa* L. (NH) *Dysphania ambrosioides*	NM	*Burkholderia* sp. and *Methylobacterium oryzae* Fungal endophyte FXZ2 *Epicoccum nigrum*	Ni, Cd Zn/Cd	Showed Ni and Cd tolerance up to 3 mm concentration FXZ2 inoculation in *Dysphania ambrosioides* induced increased Zn/Cd tolerance by exogenous production of phytohormones to promote growth, lowering oxidative damage while enhancing antioxidant properties	[140] [207]

In addition, evidence of phytoremediation of Pb by plants grown in soils contaminated by heavy metals has also been confirmed. [209, 210] reported that *Brassica napus* inoculated with *Pseudomonas fluorescens* G10 improved the total uptake of Pb from 76 to 131% of the shoot, while it was 59 to 80% (*p* < 0.05) for *Microbacterium* sp. G16, respectively. Mastretta et al. [148] supported the same finding and reported that *Sanguibacter* sp. Cd-resistant endophyte inoculated *Nicotiana tabacum* (*Solanaceae*) increased Cd concentration in shoot tissues. Yamaji et al. [257] revealed that *Clethra barbinervis* (*Clethraceae*) could tolerate high metal concentrations (Zn, 21–2600 μg/g; Cu, 2–1123 μg/g; Pb, 32–1506 μg/g) due to the support of root fungal endophytes, including *Rhizodermea veluwensis*, *Phialocephala fortinii*, and *Rhizoscyphus* sp. through K uptake promotion, growth enhancement, and decrease of heavy metal concentrations. Further studies revealed that the metal resistance mechanisms in endophytes surviving within hyperaccumulators can be attributed to activities such as metal extracellular precipitation, intracellular storage and sequestration [20, 212], conversion of hazardous metal into less or non-hazardous forms [270], and surface binding/detachment of metal [82, 130, 131].

In addition, some endophytes were isolated from different parts of non-hyperaccumulators, such as *Salix caprea* (*Salicaceae*) and *Oryza sativa* (*Poaceae*). The reported metal-resistant endophytes belonged to numerous taxa, including *Burkholderia* sp., *Methylobacterium oryzae*, *Frigoribacterium* sp., *Microbacterium* sp., and *Sphingomonas* sp. (Table 3). Kuffner et al. [115] revealed that inoculation of *Salix caprea* with *Microbacterium* sp., *Frigoribacterium* sp., *Sphingomonas* sp., and *Methylobacterium* sp. increase leaves Cd and Zn accumulation. Sharma et al. [207] concluded that seed endophytes FXZ2 inoculation in *Dysphania ambrosioides* induced increased Zn/Cd tolerance by changing Zn/Cd speciation in rhizospheric soils, as well as exogenous production of phytohormones to promote growth, lowering oxidative damage while enhancing antioxidant properties. Enhanced metal bioaccumulation in the inoculated plant was attributed to siderophores, indole acetic acid, and ACC deaminase secretion.

In general, the basic mechanism of metal adsorption involves two distinct steps: (1) passive binding/loading of metals onto the wall of dead/inactive cell without integrating energy [239] and (2) active removal (bioaccumulation), involving the movement of metals through the plasma membrane driven by energy input and followed by intracellular storage [143].

### Phytoremediation of Water and Soil Contaminated with Organic Pollutants

Industrialization and intensive agriculture are the main sources of hazardous contaminants that have deteriorated the quality of the natural ecosystem [15]. Even a small quantity of contaminants can reduce plant growth performance coupled with significant changes in soil microbe physiological processes, thus affecting critical soil biological processes [1, 139].

Phytoremediation can be used to detoxify or stabilize organic and inorganic pollutants. It is considered to be the most promising technology because it is the least disturbed at the site, cheap, and eco-friendly in nature compared to conventional remediation technologies [166, 247, 258]. Despite public acceptance, the application in the field of phytoremediation faces several obstacles, such as low biomass and slow growth, volatile contaminant evapotranspiration, and plant toxicity. Further research experiments revealed that microbe-assisted phytoremediation enhances the efficiency of phytoremediation due to its plant growth–promoting activity (e.g., siderophore production) [247]. Compared to rhizosphere microbes, endophytic microbes have been considered a better candidate for the remediation process due to their internal inhabitation that offers the opportunity to adaptation inside host cells [16, 262]. In addition, once plant growth–promoting endophytes (PGPEs) are formed in plant tissues, they are less susceptible to soil conditions’ changes but depend more on plant tissues and physiological status, such as plant health, plant growth stage, and the nutritional state [74, 191, 194, 200, 201].

Generally, the endophyte-associated phytoremediation process involves three distinct steps: (1) development, plant growth, and biomass production; (2) availability of pollutants to the host system; (3) rapid increase in endophyte population responsible for contaminant degradation. So far, many endophytic microorganisms are isolated from contaminated and non-contaminated soils capable of degrading herbicides and polyaromatic hydrocarbons pollutants (Table 4). Moore et al. (159) found that bacterial genera belonging to *Arthrobacter*, *Pseudomonas*, *Bacillus*, and *Enterobacter* recovered from different organs of poplar inhabiting near the automobile industries could remove a volatile organic compound BTEX, a component of petroleum product [159]. The mineralization of the herbicide 2,4-D was also documented by *Pseudomonas putida* VM1450 [75]. The results confirmed that 2,4-D was not detected in the soil of inoculated plants exposed to 7 or 13 mg of 2,4-D. *Pseudomonas* ITRI53 inoculated *Lolium multiflorum* var. *taurus* greatly degrades 68% of diesel-contaminated soil compared to control treatments [17]. Other bacterial endophytes such as *Achromobacter xylosoxidans* F3B and *Pantoea* sp. noted similar degradation capacity of diesel/petroleum products ITSI10 and inoculated in *Arabidopsis thaliana* and Italian ryegrass under controlled conditions, respectively [8, 90, 262]. Endophytic bacteria have also been studied to remove other aromatic compounds such as naphthalene and toluene. The inoculated pea plant with *P. putida* VM1441 (pNAH7) degraded 40% more naphthalene than the non-inoculated plant [74]. The toluene volatilization experiment suggested less toluene released from the leaves of the inoculated poplar plant with *B. cepacia* FX2 [244, 249]. Moreover, pyrene degradation increased by 43–65% in the live *Enterobacter* sp. 12J1 inoculated planted soils compared to dead bacterium inoculated planted soils [209, 210]. Furthermore, microbial species that catalyze the degradation of volatile organic contaminants, including trichloroethylene (TCE) degrading microbes, are described from *Quercus robur* (Fagaceae), *Fraxinus excelsior* (Oleaceae), and poplar growing in sites enriched with TCE [104, 248, 251]. The results of all these studies indicated that endophytic inoculation such as *B. cepacia* VM1468, *P. putida* W619-TCE, and *Enterobacter* PDN3, respectively, highly resist the release of TCE vapor in the environment, indicating the increased degradation efficiency.

**Table 4:** List of bacterial endophytes involved in the phytoremediation of organic pollutants from contaminated soil (the degradation potential of endophytes is also briefly summarized)

Site	Endophytes	Isolated plant parts	Soil contaminants	Degradation capacity	References
Field experiment; car manufacturing factory, Genk, Belgium	*Pseudomonas* sp., *Arthrobacter* sp., *Enterobacter* sp., and *Bacillus* sp.	Root, stem, and leaf	BTEX	Endophytic bacteria were isolated from the root, stem, and leaf of two cultivars of a poplar tree that grows on a BTEX-contaminated site and has the ability to degrade BTEX compounds	[159]
Campus of Institute of Technology, Carlow, Ireland	*Pseudomonas putida* VM1450	Stem sap of poplar trees	*2,4-D degradation	Inoculated plants showed a higher capacity for the removal of 2,4-dichlorophenoxyacetic acid from the soil and did not show accumulation of 2,4-dichlorophenoxyacetic acid in their aerial tissues	[75]
Diesel-contaminated site; Seibersdorf; Austria	*Pseudomonas* sp. strain ITRI53	Roots of Italian ryegrass (*Lolium multiflorum* var. *taurus*, Poaceae)	Hydrocarbon degradation	*alkB* gene could be expressed in the rhizosphere and planta. Inoculation of *Pseudomonas* sp. ITR153 was superior to the rhizosphere in colonization *alkB* expression	[17]
Greenhouse experiment; Lower Austria, Austria	*Pantoea* sp. ITSI10, *Pantoea* sp. BTRH79, and *Pseudomonas* sp. MixRI75	Italian ryegrass (*Lolium multiflorum* var. *taurus*)	Hydrocarbon (diesel) degradation	Maximum hydrocarbon reduction was reported from vegetated soil; 79% hydrocarbon reduction was achieved with inoculated plants compared to non-inoculated plants. Higher degradation potential was due to the higher microbial densities and metabolic activities of the inoculant strains	[8]
Agricultural farm of Lower Austria, Austria; greenhouse experiment	*E. ludwigii* strains ISI10-3 and BRI10-9	Italian ryegrass (*Lolium multiflorum* var. *taurus*), birdsfoot trefoil (*Lotus corniculatus* var. *leo*, Fabaceae), and alfalfa (*Medicago sativa* var. *harpe*, Fabaceae)	Hydrocarbon (diesel) degradation and ACC deaminase activities	Plants inoculated with *E. ludwigii* strains ISI10-3 and BRI10-9, highly degrade 68% of diesel-contaminated soil (spiked with 1% diesel); presence of CYP153 gene in *E. ludwigii* strains plays an important role in the degradation of pollutants	[262]
In vitro experiment; Daniaopi manmade constructed wetland, Taipei, Taiwan	*Achromobacter xylosoxidans* F3B	Roots of *Phragmites australis* (Cav.) Trin. ex Steud. (Poaceae) and *Ipomoea aquatica* Forssk. (Convolvulaceae)	Catechol and phenol (petroleum) degradation	The hydroponic test revealed 100% catechol removal by F3B inoculated *A. thaliana* compared to unplanted soil. Soil test indicated 72.7% removal of total petroleum hydrocarbons by F3B endophyte inoculated *A. thaliana* compared to unplanted soil	[90]
Microcosm experiment; Institute of Technology, Carlow, Ireland	*P. putida* VM144	Stem tissue of poplar	Naphthalene degradation	Compared to control soil, 40% more naphthalene was removed from the soil (amended with 250 mg/kg naphthalene) in the pea plant inoculated with *P. putida* VM1441(pNAH7)	[74]
Greenhouse and field trial; agricultural farm of Shanghai Normal University, China	*Burkholderia cepacia* strain FX2	*Zea mays* L. and *Triticum* sp. (Poaceae)	Toluene degradation	The toluene volatilization experiment revealed that FX2 inoculated plants release much less toluene compared to the control	[244]
Field trial experiment; Cd-contaminated site, Belgium	*Burkholderia* sp. HU001, *Pseudomonas* sp. HU002	Willow	Production of siderophores, organic acids, and indole-3-acetic acid showed increased resistance to Cd and toluene	Inoculation of both isolates in willow cutting resulted in a 80% decrease in toluene evapotranspiration without affecting the Cd uptake and translocation	[249]
In vitro experiment; Microbiological Engineering of Agricultural Environment, China; Pot Experiment	*Enterobacter* sp. 12J1	Root and stem of *Allium macrostemon* Bunge (Amaryllidaceae)	Pyrene degradation, IAA, and siderophore production	In the live bacterial inoculation experiment, an increase in pyrene removal was observed ranging from 60 to 107% in the planted soils treated with 100 mg/kg of pyrene compared to the unplanted soils. The pyrene removal rate increased by 43 to 65% in planted soils inoculated with live bacteria compared to planted soils inoculated with the dead bacteria	[209, 210]
Greenhouse experiment; Hasselt University campus; Belgium	*B. cepacia* VM1468	Yellow lupine	TCE degradation and Ni resistance	Inoculation with Ni-resistant *B. cepacia* VM1468 degrading TCE decreased the TCE release and increased the Ni uptake by the roots of the lupine plant exposed to 40 mg/L and 10 mg/L TCE	[248]
Greenhouse experiment; Hasselt University campus; Belgium	*P. putida* W619-TCE	*Populus* sp. (Salicaceae)	TCE degradation	Inoculation of *P. putida* improved TCE degradation in poplar plants exposed to 200 mg/L and 400 mg/L	[251]
In vitro experiment; University of Washington, Seattle, Washington, USA	*Enterobacter* sp. strain PDN3	*Populus* sp. (hybrid) (Salicaceae)	TCE degradation	Neither chloride released nor TCE removal was observed in samples without PDN3. However, inoculation with PDN3 reduced TCE levels from 72.4 to 30.1 µM in 24 h with a simultaneous release of 127 µM chloride ion and nearly 80% of TCE (55.3 µM) was dechlorinated by PDN3 in 5 days with the production of 166 µM chloride ion, indicating degradation capacity	[104]

In addition to soil remediation, plant endophyte associations have also been deployed to manage ground and surface water contaminated with organic contaminants (Table 5). An experimental investigation described a more than 50–70% reduction in toluene volatilization through inoculated *yellow lupine* with engineered *B. cepacia* VM 1330 compared to control plants grown in a hydroponic culture system [22]. Taghavi et al. [227] revealed that *B. cepacia* VM1468 inoculated poplar plants released five times less toluene in the air through the leaves. Furthermore, this study also concluded that horizontal gene transfer in natural endophytes could improve the phytoremediation of environmental contaminants. In addition, genetic modification of endophytes carrying foreign genes with degradation capacity has been proven to improve the phytoremediation of contaminants of aromatic and organic substances. An engineered *P. putida* W619TCE endophytic bacterium inoculated to poplar cuttings alleviated growth promotion and reduced TCE toxicity when grown in water that was contaminated with TCE [251].

**Table 5:** Bacterial endophytes involved in the phytoremediation of organic pollutants from contaminated water (a summary of the endophyte’s potential for deterioration is also provided)

Site	Host	Plant parts	Endophytes	Organic pollutants	Degradation capacity	References
In vitro experiment	Yellow lupine	Root and shoot	*B. cepacia* BU0072, *B. cepacia* VM1330, and *B. cepacia* G4	Toluene	Compared to control plants and plants inoculated with *B. cepacia* BU0072, yellow lupine inoculated with *B. cepacia* VM1330 released 50–70% less toluene in the upper compartment	[22]
In vitro experiment	*Populus* sp. (Salicaceae)		*B. cepacia* VM1468, *B. cepacia* BU61	Toluene	Poplar cuttings inoculated with *B. cepacia* VM1468 released about 5 times less toluene from the leaves compared to non-inoculated plants or plants inoculated with *B. cepacia* BU61	[227]
Greenhouse experiment; Hasselt University campus; Belgium	Yellow lupine		*B. cepacia* VM1468	TCE and Ni	Inoculation of *B. cepacia* VM1468 resulted in successful Ni uptake and reduced TCE evaporation by 90% in contaminated groundwater	[248]
Greenhouse experiment; Hasselt University campus; Belgium	*Populus* sp. (Salicaceae)		*Pseudomonas putida* W619-TCE	TCE	Inoculation of *Pseudomonas putida* W619-TCE induced plant growth promotion and reduced the phytotoxicity of TCE when grown hydroponically	[251]

Comprehensive research on endophytes proposed that the use of bacteria (preferably endophytes) that promote both plant growth and pollutant-degrading activities is superior to the use of bacteria that only promote plant growth or have pollutant-degrading activities. Therefore, an attempt is made to isolate and characterize endophytic bacteria that have plant growth–promoting and pollutant-degrading activities when growing on a contaminated site.

## Conclusion and Future Perspectives

**Fig. 7:**
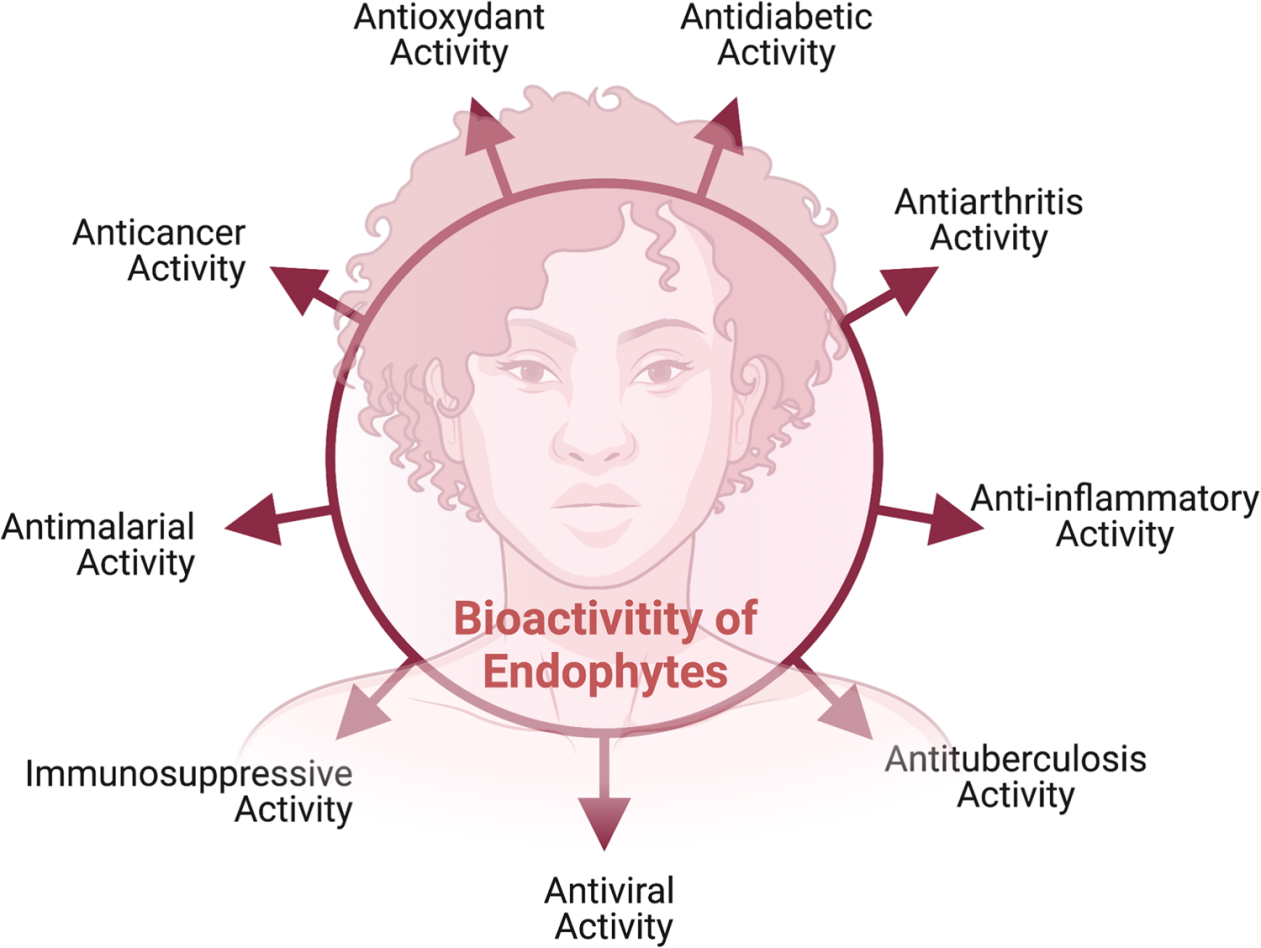
Biological activities of importance to humans present in endophytes’ metabolites. Endophytes have been reported to have the ability to produce novel metabolites which can serve as anticancer agents, glucosidase inhibitors (antidiabetic), and immunosuppressive agents; some of these endophytes also show antioxidant, antituberculosis, anti-inflammatory, and antimalarial activity, and serve as inhibitors of viruses

The application of microbial endophytes in agriculture, as well as environmental sustainability, is a growing research field. During the past two and a half decades, many studies have revealed rising interest in endophytic microbes. Endophytic microbes are known to improve host plant performance under abiotic and biotic stress conditions by altering the plants’ response. Recent advances in biotechnology and bioinformatic tools such as CRISPR (Clustered Regularly Interspaced Palindromic Repeats)–Cas system, RNA interference (RNAi), metabolomics, and next-generation sequencing systems have made the possibility of studying endophytes at the molecular level [167]. The present concept of isolation, purification, and characterization of endophytes and the research connecting biology to chemistry is now being developed. This opens new interdisciplinary dimensions and actively allows bachelor and master research students to participate in this domain of research. Research must focus on microbial endophytes to come up with new ideas to improve crop productivity on a pilot scale. Endophytes play an important role in producing a wide variety of naturally occurring secondary metabolites (such as tyrosol, saadamycin, and munumbicins) showing the industrial application in pharmaceutics and thus human health. In this regard, researchers from all over the world are continuously exploring hidden endophytic microbes for novel potent bioactive compounds that can be used as potential therapeutics. Figure 7 shows the importance of the biological activities of endophytic metabolites. Endophytes are reported to be a warehouse of new metabolites that can be widely used as antimicrobial, anticancer, immunosuppressant, antiarthritic, and anti-insect drugs. Although several bioactive compounds produced by endophytes, such as camptothecin, vinblastine, hypericin, and podophyllotoxin, have already been commercialized, novel bioactive compounds seem promising in the case of most pathogenic microorganisms in overcoming the problem of antibiotic resistance.

Taken together, new bioactive compounds emitted by endophytes, particularly endophytic actinomycetes, could make a significant contribution to the current and future challenges of agriculture, the environment, and medicine. To isolate and characterize new endophytes with specific features that could be useful for crop production, comprehensive bioprospecting research of endophytic microbes from various ecological niches (e.g., harsh habitats, the marine environment, etc.) is required. We anticipate a shift in practice in the future, with a greater emphasis on optimizing the interaction between plants and soil microorganisms and endophytes. However, molecular mechanisms that explain the interaction between plants and endophytes have yet to be discovered. They will open a new door to the isolation and characterization of new molecules for humans and provide a new way to improve crops and environmental sustainability.

## Data Availability Statement

This is a review article. So, all the data are taken/extracted from the cited references or are furnished in the manuscript at the relevant place. The data that support the present study are available in the cited references.
